# Association between masticatory function, frailty, and functional disability: an observational study

**DOI:** 10.1186/s12877-024-05131-w

**Published:** 2024-06-21

**Authors:** Hiroko Furuhashi, Takanori Honda, Yoshihiko Furuta, Shoko Tomooka, Takahiro Tajimi, Yasumi Kimura, Daigo Yoshida, Toshiharu Ninomiya

**Affiliations:** 1 Department of Epidemiology and Public Health, Graduate School of Medical Sciences, Kyushu University, Maidashi 3-1-1, Higashi-Ku, Fukuoka City, 812-8582 Japan; 2 Center for Cohort Studies, Graduate School of Medical Sciences, Kyushu University, Fukuoka, Japan; 3 Department of Medicine and Clinical Science, Graduate School of Medical Sciences, Kyushu University, Fukuoka, Japan; 4 Section of Geriatric Dentistry and Perioperative Medicine in Dentistry, Division of Maxillofacial Diagnostic and Surgical Sciences, Faculty of Dental Science, Kyushu University, Fukuoka, Japan; 5 Department of Orthopaedic Surgery, Graduate School of Medical Sciences, Kyushu University, Fukuoka, Japan; 6 Emergency and Critical Care Center, Kyushu University Hospital, Fukuoka, Japan; 7 Department of Health Nutrition, Faculty of Health Sciences, Hiroshima Shudo University, Hiroshima, Japan; 8 Division of Community Health Nursing and Home Care Nursing, Graduate School of Nursing, Fukuoka Nursing College, Fukuoka, Japan

**Keywords:** Long-term care, Functional disability, Frailty, Oral function, Mastication

## Abstract

**Background:**

Increase in functional disability in aging societies is an international medical and public health issue. Masticatory function may be a potential risk factor for functional disability, but the role of frailty in the association has not been clarified.

**Methods:**

Forty thousand five hundred sixty-two community-dwelling older adults aged 65 years and over who were insured by public health insurance as of April 2018 were followed up for a median of 3.0 years. Masticatory function was categorized as good, moderate, or poor based on a self-reported questionnaire. The development of functional disability was defined as a new certification of the need for long-term care. A Cox proportional hazards model was used to calculate hazard ratios (HRs) and their 95% confidence intervals (CIs).

**Results:**

During the follow-up period, 1,397 individuals experienced functional disability. After adjusting for age, sex, comorbidities, medical history, and lifestyle behaviors, the HR for incident functional disability was significantly higher in the moderate and poor groups compared to the good group (moderate, HR 1.21 [95% CI, 1.07–1.37]; poor, HR 1.64 [95% CI, 1.03–2.62]). However, after additional adjustment for frailty-related factors—namely, underweight, regular exercise, and gait speed—the association was attenuated in both the moderate group (HR 1.06 [95% CI, 0.94–1.21]) and the poor group (HR 1.51 [95% CI, 0.94–2.41]).

**Conclusions:**

Masticatory dysfunction was significantly associated with incident functional disability in a community-dwelling older Japanese population. Our findings suggest that masticatory dysfunction may be a surrogate of frailty rather than a direct cause of functional disability.

**Supplementary Information:**

The online version contains supplementary material available at 10.1186/s12877-024-05131-w.

## Background

Functional disability has become a major medical and public health issue due to the global growth in the older population. About 20% of older adults have functional disability [1–3], and functional disability has been reported to induce death [4] and raise the expense of long-term care (LTC) in society [5–7].

Recently, oral health has attracted attention as a factor associated with functional disability [8–19]. Previous studies have reported that a decreased number of teeth as an indicator of oral health status was associated with functional disability [8–12]. After the number of teeth, masticatory function has been reported to account for the next-highest population-attributable fraction of incident functional disability [8]. However, the epidemiological evidence has been inconsistent, with some studies reporting that masticatory dysfunction was associated with functional disabilities [8, 14, 15] and others finding no association or an age-dependent association [16, 17]. We hypothesized that the discrepancy might be attributable to frailty-related factors. Indeed, because masticatory dysfunction has been increasingly recognized as a factor relevant to frailty [20–22], frailty might explain the association between masticatory dysfunction and functional disability. However, the influence of frailty on the association between masticatory function and functional disability remains unclear.

This study aimed to examine the association between masticatory function and the risk of developing functional disability, taking frailty-related factors into account, by using an extensive administrative database from a Japanese urban area.

## Methods

### Study design

**Fig. 1:**
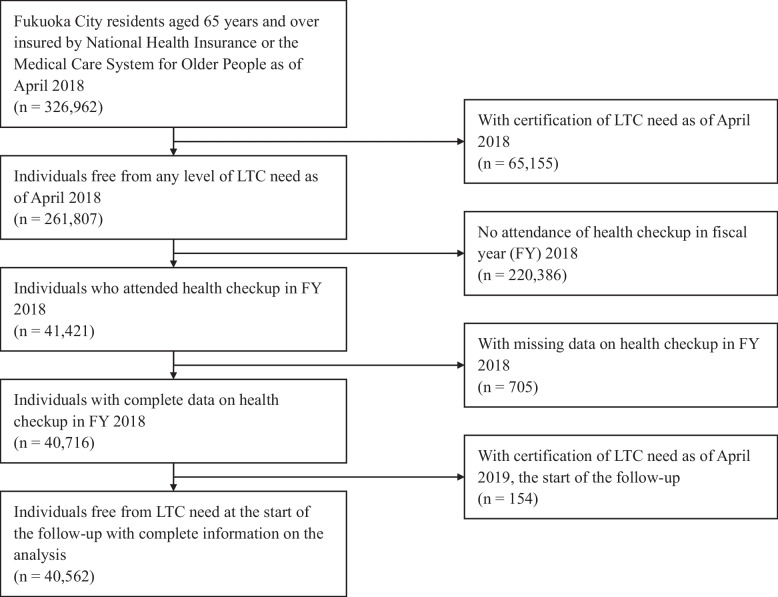
Flow diagram of participants

This study is a retrospective cohort study with secondary use of data provided by Fukuoka City [23]. We used information on demographics, health checkups, and LTC insurance. Participants were Fukuoka City residents aged 65 years and over who were insured by National Health Insurance or the Medical Care System for Older People as of April 2018. Among them, we excluded those who had not attended health checkups, those certified as having any level of LTC need, and those with missing data on variables of interest in fiscal year (FY) 2018. In addition, those certified as having any level of LTC need by April 2019, the start month of observation, were excluded from the analysis. As of April 2018, 326,962 Fukuoka City residents aged 65 years and over were insured by National Health Insurance or the Medical Care System for Older People. Among them, the following individuals were excluded: 65,155 persons certified as having any level of LTC need as of April 2018; 220,386 persons who had not received health checkups in FY 2018; 705 persons who had missing data on the results of the health checkups; and 154 persons certified as having any level of LTC need by the start of observation in April 2019 (Fig. 1).

### Definition of masticatory function

The masticatory function was assessed subjectively in FY 2018 by the following question: "Which of the following best describes your state when you chew a meal?" The participants were categorized into three groups based on their responses: "I can chew and eat anything" (good), "I have difficulty chewing due to concerns about teeth, gums, and bite" (moderate), and "I can hardly chew" (poor).

### Definition of the development of functional disability

The development of functional disability was defined as a new certification of the need for LTC at care need level 1 or higher and is coded as a binary variable (yes/no) [24, 25]. Eligibility of the need for LTC is screened algorithmically by a questionnaire based on activities of daily living, and certification of the need for LTC is finally determined by an expert committee consisting of healthcare professionals [24, 25]. There are seven levels of the certification: support levels 1 and 2, for individuals who require assistance with daily living but are not considered to have a disability; and care need levels 1 (most minor disability) to 5 (most severe disability), for individuals who require continuous care. The LTC certification has been reported to correlate with the Barthel index [26] and is commonly used in recent studies to define functional disability [27, 28]. For participants for whom data were available at baseline, cases of new certification requiring LTC, moving out from the city, and death between April 2019 and March 2022 were identified.

### Covariates

The following covariates were used for analysis: age, sex, underweight, presence of comorbidities defined by health checkup results (hypertension, diabetes, and dyslipidemia), self-reported medical history (cerebrovascular disease, heart disease, and kidney disease or dialysis), and self-reported lifestyle behaviors (current smoking, daily drinking, regular exercise, and gait speed). Underweight was defined as body mass index < 18.5 kg/m^2^. Hypertension was defined as systolic blood pressure ≥ 140 mmHg, diastolic blood pressure ≥ 90 mmHg, or taking antihypertensive agents. Diabetes mellitus was defined as a fasting blood glucose level ≥ 126 mg/dL, HbA1c ≥ 6.5%, or taking glucose-lowering agents. Dyslipidemia was defined as serum triglycerides ≥ 150 mg/dL, serum high-density lipoprotein cholesterol < 40 mg/dL, serum low-density lipoprotein cholesterol ≥ 140 mg/dL, or taking lipid-lowering agents. The following question assessed regular exercise: "Are you in the habit of exercising to sweat lightly for over 30 min a time, two times weekly, for over a year?" Gait speed was assessed by asking "Is your walking speed faster than the speed of those of your age and sex?" Among these covariates, underweight, no regular exercise, and slow gait speed were considered as frailty-related factors.

### Statistical analysis

We compared age and sex between individuals included in and individuals excluded from the present analysis among those free from any level of LTC need as of April 2018. We only compared age and sex because other covariates obtained in health checkups were unavailable. In addition, we examined the differences in the available covariates between the included and the excluded participants among the health checkup attendees. We used the logistic regression model for categorical variables and the linear regression model for continuous variables for trend tests of baseline characteristics according to the masticatory function [29]. A Cox proportional hazards model was used to calculate the hazard ratios (HRs) and 95% confidence intervals (CIs) for the risk of developing functional disability conferred by the masticatory function. In the multivariable model, the following covariates were included: age, sex, comorbidities (hypertension, diabetes, or dyslipidemia), medical histories (cerebrovascular disease, heart disease, kidney disease, or dialysis), and lifestyle behaviors (smoking and alcohol consumption). Frailty-related factors (underweight, regular exercise, and gait speed) were additionally adjusted to examine the influence of these factors in the association of masticatory function with functional disability. We calculated the percentage reduction in effect size in the linear scale (i.e., natural log of hazard ratio) after adding frailty-related factors using the following formula: 100*[ln(HR_model1_)- ln(HR_model2_)]/ ln(HR_model1_). Subgroup analyses by frailty-related factors were performed and the heterogeneity was tested using a likelihood ratio test. All analyses were performed using SAS version 9.4 (SAS Institute, Cary, NC), and *p*-values less than 0.05 were considered statistically significant.

## Results

A total of 40,562 persons were eligible for the analysis. Among those free from any level of LTC need as of April 2018, those included in the present analysis were significantly younger and more likely to be female than those excluded for lack of other clinical information (Additional Table 1 in Additional file). Among those who underwent health checkups, those included in the final analysis only differed in the proportion of dyslipidemia compared to those excluded (Additional Table 2 in Additional file). Table 1 shows the participants' characteristics at baseline by masticatory function.

**Table 1:** Baseline characteristics by masticatory function

	Masticatory function			*P*-value for trend
	Good (*n* = 32,611)	Moderate (*n* = 7,720)	Poor (*n* = 231)	
Age, years	70.8 (4.3)	70.9 (4.4)	71.2 (5.1)	0.02
Male, %	41.7	44.2	61.0	< 0.001
Hypertension, %	50.3	51.7	58.4	0.004
Diabetes, %	11.6	13.2	19.9	< 0.001
Dyslipidemia, %	63.0	62.2	61.0	0.17
History of cerebrovascular disease, %	3.8	4.8	10.4	< 0.001
History of heart disease, %	6.9	7.9	13.4	< 0.001
History of kidney disease or dialysis, %	0.7	0.8	1.3	0.14
Current smoking, %	8.6	14.9	26.0	< 0.001
Daily drinking, %	25.1	26.6	25.1	0.01
***Frailty-related factors***				
Underweight, %	7.7	9.5	6.9	< 0.001
No regular exercise, %	45.2	54.8	64.1	< 0.001
Slow gait speed, %	36.3	48.5	54.5	< 0.001

Data are presented as the mean value (standard deviation) for age and percentages for other variables. *P*-values were determined using a regression model for age and logistic regression models for other categorical variables

During a median observation period of 3.0 years (range 0.08 to 3.0 years), 1,397 individuals developed functional disability. Table 2 shows the HRs and the 95% CIs for developing functional disability by masticatory function. There was a significant increase in the HR for the moderate and the poor masticatory function group compared to the good group in the age- and sex-adjusted model (HR 1.26 [95% CI, 1.11–1.42] for the moderate group; HR 1.96 [95% CI, 1.23–3.12] for the poor group) and in the multivariable model (HR 1.21 [95% CI, 1.07–1.37] for the moderate group; HR 1.64 [95% CI, 1.03–2.62] for the poor group). In contrast, no significant increase in HR was observed after adjusting for frailty-related factors in the moderate group (HR 1.06 [95% CI, 0.94–1.21]) or the poor masticatory function group (HR 1.51 [95% CI, 0.94–2.41]). The percentage reduction in effect size was 67.5% in the moderate group and 17.4% in the poor group, respectively. No significant heterogeneity was found in subgroup analyses for age category, sex, and frailty-related factors (Table 3).

**Table 2:** Hazard ratios (95% confidence intervals) of masticatory function for functional disability

Masticatory function	No. of events/individuals	Age- and sex-adjusted model		Multivariable-adjusted model 1^a^		Multivariable-adjusted model 2^b^	
		HR (95% CI)	*P*-value	HR (95% CI)	*P*-value	HR (95% CI)	*P*-value
Good	1,055/32,611	1.00 (Reference)		1.00 (Reference)		1.00 (Reference)	
Moderate	324/7,720	1.26 (1.11–1.42)	< 0.001	1.21 (1.07–1.37)	0.003	1.06 (0.94–1.21)	0.34
Poor	18/231	1.96 (1.23–3.12)	0.005	1.64 (1.03–2.62)	0.04	1.51 (0.94–2.41)	0.09

*Abbreviations:* *HR* Hazard ratio, *CI* Confidence interval

^a^Adjusted for age, sex, hypertension, diabetes, dyslipidemia, history of cerebrovascular disease, heart disease, kidney disease or dialysis, current smoking, and daily drinking

^b^Adjusted for covariates included in the multivariable-adjusted model 1 plus frailty-related factors

**Table 3:** Hazard ratios (95% confidence interval) for functional disability by frailty-related factors

	No. of events/individuals	Masticatory function					*P*-value for heterogeneity
		Good	Moderate		Poor		
		HR (95% CI)	HR (95% CI)	*P*-value	HR (95% CI)	*P*-value	
Age							
65–74 years	828/34,551	1.00 (Reference)	1.14 (0.97–1.34)	0.11	1.68 (0.92–3.06)	0.09	0.51
75 years and over	569/6,011	1.00 (Reference)	1.00 (0.82–1.22)	0.99	1.99 (0.93–4.25)	0.08	
Sex							
Male	705/17,155	1.00 (Reference)	1.07 (0.89–1.27)	0.47	1.59 (0.89–2.83)	0.11	0.97
Female	692/23,407	1.00 (Reference)	1.03 (0.86–1.24)	0.75	1.39 (0.62–3.12)	0.43	
Underweight							
No	1,202/37,293	1.00 (Reference)	1.08 (0.94–1.24)	0.29	1.46 (0.87–2.43)	0.15	0.88
Yes	195/3,269	1.00 (Reference)	1.03 (0.75–1.41)	0.88	2.07 (0.61–7.01)	0.24	
Regular exercise							
No	760/19,119	1.00 (Reference)	1.11 (0.95–1.31)	0.19	1.55 (0.91–2.66)	0.11	0.60
Yes	637/21,443	1.00 (Reference)	0.99 (0.81–1.21)	0.91	1.36 (0.51–3.64)	0.55	
Gait speed							
Slow	820/15,701	1.00 (Reference)	1.09 (0.93–1.28)	0.27	1.67 (0.96–2.91)	0.07	0.60
Fast	577/24,861	1.00 (Reference)	1.00 (0.80–1.24)	0.99	1.08 (0.44–2.63)	0.87	

Models were adjusted for age, sex, hypertension, diabetes, dyslipidemia, history of cerebrovascular disease, heart disease, kidney disease or dialysis, current smoking, daily drinking, and frailty-related factors other than the stratification factor

*Abbreviations:* *HR* Hazard ratio, *CI* Confidence interval

## Discussion

Using extensive data from more than 40,000 older adults, the present study demonstrated that masticatory dysfunction was significantly associated with the development of functional disability, while this statistically significant association disappeared after adjusting for frailty-related factors. None of the subgroup analyses of age, sex, underweight, regular exercise, or gait speed showed significant heterogeneity. These findings suggested that frailty-related factors might explain the association between masticatory dysfunction and incident functional disability.

Previous studies showed a significant association between masticatory dysfunction and functional disability [8, 14, 15]. These findings were consistent with ours. In contrast, this significant association was attenuated after fully adjusting for frailty-related factors. A similar finding was reported in a study by Aida et al. [16], in which BMI and walking activity were considered as covariates, suggesting that frailty could explain the association between masticatory dysfunction and functional disability. Frailty is a medical syndrome characterized by decreased resilience and reserves, in which a mutually exacerbating cycle of declines in multiple domains leads to physiologic dysregulation [30]. As far as we know, there was no studies addressed the potential role of the syndromic condition of frailty in the link between oral health and functional disability. Unfortunately, we cannot determine the temporal relationship between masticatory function and frailty, as these variables were measured simultaneously. This constraint of the study design precluded us from verifying if masticatory dysfunction preceded frailty-related factors (i.e., mediation) or if frailty-related factors preceded or correlated with masticatory dysfunction (i.e., confounding). Future detailed investigations on the relationship are warranted to elucidate the potential causal mechanisms. 

Previous studies have proposed several possible mechanisms for the association between masticatory function and functional disability, including pathways mediated by nutrition [31, 32], inflammation [33, 34], and social interactions [35, 36]. However, the attenuation of the association between masticatory function and functional disability after adjusting for frailty-related factors suggests that masticatory function is unlikely to be directly related to functional disability. It is plausible that frailty-related factors are involved in the form of a mediator or a confounder in the association between masticatory function and functional disability. Unfortunately, the causal pathway between these factors could not be clarified in this study. Nevertheless, it seems certain that subjective masticatory dysfunction reflects the presence of frailty-related factors, and thus, it may be useful as a surrogate measure of underlying frailty status.

In addition, we found no evidence of heterogeneity across frailty-related factors as well as age, suggesting that these factors might not modify the influence of masticatory function. A previous study suggested that the association between masticatory dysfunction and functional disability may differ by age group [17], but this was not replicated in the present study. By taking frailty-related factors into account and using a larger sample size, this study may provide robust evidence of the lack of association across the baseline status.

The strengths of our study include its large sample size, which allowed for high statistical power, and its consideration of a broad range of covariates. Limitations should also be noted. First, the masticatory function was assessed subjectively, possibly leading to misclassification. Second, frailty-related factors only included three indicators: underweight, no regular exercise, and slow gait speed: this may be insufficient to define physical frailty based on the conventional definition. Third, this study included only individuals who had undergone health checkups, and therefore, the study participants may have been more health-conscious than the general population. In this regard, however, there was no evidence of systematic differences in health status between those who were included in and excluded from the final analysis (see Additional Table 1, 2 in Additional file), which could partly support the applicability of our findings to other populations who underwent health checkups. Fourth, the observation period was relatively short (3 years). A previous study demonstrated that decreased occlusal support and tooth loss influenced the risk of functional disability across a relatively long-term follow-up (> 3 years) but not over a shorter term, suggesting that it may take time for the association to become apparent [11]. Future prospective studies over a more extended period are warranted. Finally, since this study only included residents of one ordinance-designated city in Japan, generalizability to other regions is limited.

## Conclusions

Using an extensive administrative database of Japanese urban residents, masticatory dysfunction was associated significantly with the development of functional disability, and this statistically significant association disappeared after adjusting for frailty-related factors. Our findings suggested that masticatory dysfunction may be a surrogate of frailty rather than a direct cause of functional disability.

### Supplementary Information

Additional file 1: Additional tables. Comparison of baseline characteristics between individuals included in and excluded from the present analysis.

## Data Availability

The datasets generated and analysed during the current study are not publicly available due to the contract that not allowed to publish individual data externally, but summary information would be available from the corresponding author on reasonable request.
