# Supplementary material for: Association between masticatory function, frailty, and functional disability: an observational study
Source: BMC Geriatr. 2024 Jun 21;24:538. doi: 10.1186/s12877-024-05131-w (PMC11193275; doi:10.1186/s12877-024-05131-w)
Supplement: Supplementary file 1 — Additional file 1: Additional tables. Comparison of baseline characteristics between individuals included in and excluded from the present analysis. [file 12877_2024_5131_MOESM1_ESM.docx]

**Additional Table 1. Comparison of age and sex between individuals included in and excluded from the present analysis among 261,807 individuals free from any level of LTC need as of April 2018**

|  | Excluded (n = 221,245) | Included (n = 40,562) | *P*-value |
| --- | --- | --- | --- |
| Age, years | 73.7 (6.7) | 70.8 (4.3) | < 0.001 |
| Male, % | 44.9 | 42.3 | < 0.001 |

Data are presented as the mean value (standard deviation) for age and percentages for male. P-values were determined using a t-test for age and a chi-squared test for male.

**Additional Table 2. Comparison of baseline characteristics between individuals included in and excluded from the present analysis among 41,421 individuals who underwent health checkups**

|  | Excluded (n = 859) | Included (n = 40,562) | *P*-value |
| --- | --- | --- | --- |
| Age, years | 73.9 (5.5) | 70.8 (4.3) | < 0.001 |
| Male, % | 44.4 | 42.3 | 0.23 |
| Hypertension, % | 50.8 | 50.6 | 0.93 |
| Diabetes, % | 11.5 | 12.0 | 0.70 |
| Dyslipidemia, % | 57.9 | 62.8 | 0.003 |
| History of cerebrovascular disease, % | 4.9 | 4.0 | 0.19 |
| History of heart disease, % | 5.4 | 7.1 | 0.06 |
| History of kidney disease or dialysis, % | 0.8 | 0.7 | 0.72 |
| Current smoking, % | 10.6 | 9.9 | 0.51 |
| Daily drinking, % | 25.1 | 25.3 | 0.88 |
| ***Frailty-related factors*** |  |  |  |
| Underweight, % | 7.2 | 8.1 | 0.37 |
| No regular exercise, % | 48.1 | 47.1 | 0.62 |
| Slow gait speed, % | 42.6 | 38.7 | 0.06 |

Data are presented as the mean value (standard deviation) for age and percentages for other variables. P-values were determined using a t-test for age and chi-squared tests for other categorical variables. Missing values for each variable were excluded.
